# Natural CLA-Enriched Lamb Meat Fat Modifies Tissue Fatty Acid Profile and Increases n-3 HUFA Score in Obese Zucker Rats

**DOI:** 10.3390/biom9110751

**Published:** 2019-11-19

**Authors:** Gianfranca Carta, Elisabetta Murru, Claudia Manca, Andrea Serra, Marcello Mele, Sebastiano Banni

**Affiliations:** 1 Department of Biomedical Sciences, University of Cagliari, 09042 Monserrato, CA, Italy; m.elisabetta.murru@gmail.com (E.M.); claumanca@hotmail.com (C.M.); banni@unica.it (S.B.); 2 Department of Agriculture, Food and Environment, University of Pisa, 56124 Pisa, Italy; andrea.serra@unipi.it (A.S.); marcello.mele@unipi.it (M.M.)

**Keywords:** CLA, conjugated linoleic acid, ALA, α-linolenic acid, n-3 HUFA score, meat fat, vegetable fat

## Abstract

Ruminant fats are characterized by different levels of conjugated linoleic acid (CLA) and α-linolenic acid (18:3n-3, ALA), according to animal diet. Tissue fatty acids and their N-acylethanolamides were analyzed in male obese Zucker rats fed diets containing lamb meat fat with different fatty acid profiles: (A) enriched in CLA; (B) enriched in ALA and low in CLA; (C) low in ALA and CLA; and one containing a mixture of olive and corn oils: (D) high in linoleic acid (18:2n-6, LA) and ALA, in order to evaluate early lipid metabolism markers. No changes in body and liver weights were observed. CLA and ALA were incorporated into most tissues, mirroring the dietary content; eicosapentaenoic acid (EPA) and docosahexaenoic acid (DHA) increased according to dietary ALA, which was strongly influenced by CLA. The n-3 highly-unsaturated fatty acid (HUFA) score, biomarker of the n-3/n-6 fatty acid ratio, was increased in tissues of rats fed animal fats high in CLA and/or ALA compared to those fed vegetable fat. DHA and CLA were associated with a significant increase in oleoylethanolamide and decrease in anandamide in subcutaneous fat. The results showed that meat fat nutritional values are strongly influenced by their CLA and ALA contents, modulating the tissue n-3 HUFA score.

## 1. Introduction

Dietary fats are often associated with diet-derived health problems such as obesity, coronary heart disease, diabetes, and tumors. Nevertheless, some dietary fatty acids (FAs) have been found to act as preventing factors against cardiovascular disease (CVD) [1] and certain types of tumors [2,3]. The unusual fatty acid, conjugated linoleic acid (CLA) (CD18:2), and n-3 polyunsaturated fatty acids (PUFAs) are considered the major preventing factors that are naturally present in food derived from milk and meat ruminant fats, and from fish, respectively. Consequently, since ruminant products rich in saturated fatty acids (SFAs) are not entirely acceptable to consumers [4], strategies to manipulate the fat content and fatty acid (FA) composition of ruminant meat and milk have been proposed [5,6]. Natural CLA, mainly cis-9, trans-11 CLA (18:2c9,t11), a conjugated dienoic isomer of linoleic acid (18:2n-6, LA), derives from the incomplete biohydrogenation of LA in the rumen, and/or by the action of stearoyl-CoA desaturase (∆9 desaturase) on vaccenic acid (trans-11 18:1, VA) within the mammary gland [7,8]. Natural CLA and its isomers constitute a special category of trans FAs that have been shown to exert anti-carcinogenic, anti-obesity, and anti-inflammatory effects, among others [2,9,10], by interfering with the metabolism of n-6 PUFAs [11,12]. It is possible to increase the content of CLA in meat and milk from ruminants when animals graze fresh pastures [13], and through supplementation of the diet with oils or seeds [14,15], like safflower oil, which is rich in LA [16], or linseed oil, which is high in α-linolenic acid (18:3n-3, ALA) [17]. CLA is accumulated in a similar fashion as oleic acid (18:1, OA), and desaturated and elongated in tissues to conjugated linolenic acid (CD18:3) and conjugated eicosatrienoic acid (CD20:3), retaining an unaltered conjugated diene structure [18]. CLA is also efficiently β-oxidized in peroxisomes, and acts as avid ligand of peroxisome proliferator-activated receptors type α (PPAR-α) [19], which regulate the expression of genes involved in peroxisomal β-oxidation [20].

The main n-3 long-chain, highly-unsaturated FAs (HUFAs) are represented by eicosapentaenoic acid (20:5n-3, EPA) and docosahexaenoic acid (22:6n-3, DHA), which exert anti-inflammatory and hypolipidemic effects through increased PPAR-α-mediated β-oxidation of FAs [21,22]. EPA and DHA may derive in tissues from seafood products or from the elongation and desaturation process of ALA. However, the conversion rate of ALA to DHA is too low to be considered efficient from a nutritional point of view [23]. Therefore, there is a need to increase the intake of foods with n-3 PUFAs [24].

The balance of LA and ALA, as precursors of the n-6 and n-3 FA families, is critical for the formation of n-3 HUFA [25]. It has been shown that a maximal incorporation of DHA into tissues can be achieved using diets with LA/ALA ratios between 4:1 and 2:1 [26,27,28]. Lands et al. measured the n-3 HUFAs content of red blood cells and tissues in rats consuming different LA/ALA ratios, and developed the concept that diets high in LA would inhibit the synthesis of n-3 HUFAs by simple competitive inhibition of the ∆6 desaturase (∆6-D) enzyme and other enzymes [29,30].

The n-3 HUFA score that is obtained as the percentage of n-3 highly-unsaturated fatty acids (HUFA ≥ 20 carbons and ≥ 3 double bonds) in the total HUFAs pool is a potential blood biomarker of n-3 FAs intake and tissue status. HUFAs are mainly incorporated into phospholipids (PLs), and are potential precursors of biologically-active eicosanoids and docosahexaenoids [31]. Because the n-3 HUFA score has been shown to be less variable than n-3 FAs in the blood and tissues of rats, it could serve as a modifiable risk factor for CVD [31].

The ratio between the sum of n-3 HUFAs (EPA, DHA) + dihomo-γ-linolenic acid (20:3n-6, DGLA) and arachidonic acid (20:4n-6, AA), i.e., the anti-inflammatory FA index (AIFAI), provides a marker of the ability to decrease the formation of n-6 eicosanoids [32,33]. In fact, AIFAI has been reported to increase in association with a significant decrease in the formation of PGE2, 6-keto-PGF, prostanoids, and TNFα [34].

Many studies have found that a high fat diet can induce obesity, implying the obesogenic role of dietary fat; however, most of these studies did not take into account the fact that dietary FA composition is crucial in the regulation of body fat deposition and distribution. Some authors have described obesity resistance and reduced hypertrophy of visceral fat pads when employing fish oil-based diets. This might be related to increased lipid oxidation in these animals due to the n-3 HUFA-induced activation of PPAR-α [35,36]. Similarly, dietary CLA has been shown to decrease body fat in animals and humans [37]. Some of the effects of dietary FAs have been shown to be mediated by endocannabinoids (EC), namely anandamide (AEA) and 2-arachidonoyl–glycerol (2-AG), both of which are derived from AA, and by AEA congeners such as *N*-palmitoylethanolamide (PEA) and *N*-oleoylethanolamide (OEA), which are avid ligands of PPAR-α. An overactive endocannabinoid system may favor visceral fat deposition and thereby obesity, while the activation of PPAR-α has been shown to reduce body weight [35,36].

In the present study, we aimed to evaluate whether the peculiar nutritional effect of CLA in combination with ALA on increasing n-3 HUFA score, found in humans with dietary CLA-enriched cheese [38,39], would also be confirmed with dietary lamb meat fats which were differentially enriched in CLA and ALA compared to vegetable fats in Zucker rats, a rat model of obesity [40].

## 2. Materials and Methods 

### 2.1. Reagents

The acetonitrile, methanol, chloroform, n-hexane, ethanol, acetic acid, and fatty acids standards were HPLC grade, and like deferoxamine mesylate, were purchased from Sigma Chemicals Co. (St. Louis, MO, USA). Ascorbic acid, potassium hydroxide, and hydrochloric acid were purchased from Carlo Erba (Milano, Italy). Internal deuterated standards for the AEA, 2-AG, and OEA quantification by isotope dilution ([^2^H]_8_ AEA, [^2^H]_5_ 2-AG, [^2^H]_2_ OEA) were purchased from Cayman Chemicals (MI, USA).

### 2.2. Animals and Diets

Twenty-four male obese Zucker rats (Harlan) four weeks of age with an initial weight of 200 ± 15 g were randomly assigned to four groups, and fed for four weeks with different diets containing 6% total fat, which provided 14% of the total energy (%en). The diets, based on the AIN-93G formulation with the substitution of soybean oil with experimental fats, differed only for FAs composition: (A) fat enriched in CLA, 1.3 g/kg of total diet, obtained from the meat of suckling milk from grazing ewes; (B) fat enriched in ALA and low in CLA, respectively 0.9 and 0.6 g/kg of the total diet, obtained from the meat of heavy lambs fed a diet based on cereal grains and integrated with rolled linseed + stoned olive cake; (C) fat containing LA, ALA, and CLA, respectively 2.3, 0.3, and 0.4 g/kg of total diet, obtained from the meat of lambs fed a diet based on cereal grains; (D) fat high in LA, and ALA, respectively 13.1 and 0.6 g/kg of total diet, of vegetable origin from a mixture of olive and corn oils, as depicted in Table 1. Diets A and B were characterized by a high content of trans FAs (VA and CLA), and ALA. The SFA content was higher in animal fat, while unsaturated fatty acids were higher in the diet with vegetable fat. Animal fat for the diets was obtained from the carcasses of lambs produced according to the feeding protocols described by Serra et al. [41] for diet A and Mele et al. [42] for diets B and C. The diets were prepared by Harlan.

Body weight and food intake were measured weekly across the study. Body length, from tip of nose to the base of the tail, was measured at baseline (week 0), and at the study endpoint (week 4).

All experiments were performed according to the guidelines and protocols approved by the European Union (EU Council 86/609; D.L. 27.01.1992, no. 116) and by the Animal Research Ethics Committee of the University of Cagliari, Italy. The authorization number from the Italian Ethical Committee, approved on 28 September 2018, is 733/2018-P.

### 2.3. Tissues and Blood Sampling

Before sacrifice, rats were fasted for 12 h. After Fentanyl treatment (100 µg/kg of body weight) rats were euthanized without any further anesthesia by decapitation. Immediately after death, liver, heart, hypothalamus, visceral and subcutaneous adipose tissues were taken and stored at −80 °C. Blood was taken and centrifuged at 2000× *g* for 15 min at room temperature, plasma was stored at −80 °C for future lipid analyses.

### 2.4. Lipid Analyses

Fatty acid analysis was conducted from the total lipids previously extracted from tissues by the method of Folch [43]. Aliquots of chloroform were dried and mildly saponified as previously described [44] in order to obtain free fatty acids for HPLC analysis. The separation of unsaturated fatty acids was carried out with an Agilent 1100 HPLC system (Palo Alto, CA, USA) equipped with a diode array detector, as previously reported [45]. Since SFAs are transparent to UV detection, they were measured, after methylation, by Agilent 6890 gas chromatography (Palo Alto, CA, USA), as described in [46].

Endocannabinoid and congener quantification is described in [47]. Deuterated EC and congeners were added as internal standards to the samples before extraction. Analyses were carried out by liquid chromatography, atmospheric pressure chemical ionization, and MS (LC–APCI–MS) (Palo Alto, CA, USA), using selected ion monitoring (SIM) at M+1 values for the compounds and their deuterated homologs.

The n-3 HUFA score was calculated as the percentage of the sum of n-3 FAs with 20 or more carbon atoms and three or more double bonds, divided by the sum of total FAs with 20 or more carbon atoms and more than three double bonds [31]:

n-3 HUFA score = (EPA + DHA + docosapentaenoic acid (22:5n-3, DPAn-3))/(EPA + DHA + DPAn-3 + DGLA + AA + 22:4n-6 + DPAn-6 + 20:3n-9) × 100(1)

The anti-inflammatory FA index is obtained as [33]:

AIFAI = (EPA + DHA + DGLA)/(AA × 100)(2)

### 2.5. Statistical Analysis

Data are expressed as the mean ± SEM, specifically, fatty acids as nmoles per gram of tissue or ml of plasma, or g/kg diet. EC and congeners are expressed as Mol% compared to total FAs. Multiple unpaired comparison tests were performed by ordinary one-way ANOVA followed by a Tukey’s posthoc multiple comparison test in order to check the effect of specific dietary lipids on lipid metabolism in an animal model of obesity. The statistical analyses were performed using the GraphPad Prism 6.01 Software (La Jolla, CA, USA).

## 3. Results

No variations of food intake, body and liver weights, or liver total lipid concentration were detected in the obese Zucker rats in relation to different dietary fat sources. The BMI of rats fed diet D (0.96 ± 0.022), containing vegetable fat, was slightly, but not significantly increased compared to rats fed diet C (0.89 ± 0.023).

### 3.1. Tissue FA Profile 

An analysis of FAs revealed that the tissue FA profiles, except in the hypothalamus, were strongly influenced by dietary FAs (Table 2). As expected, CLA concentrations mirrored the diet CLA content in the following order A > B > C > D in liver, subcutaneous adipose tissue (SAT), and visceral adipose tissue (VAT) (Table 2). The same pattern was observed in plasma, even though there were not significant differences between diets B and C or C and D; in heart, CLA reached similar concentrations in the A and B groups, i.e., higher than diets C and D, while, as anticipated, no changes were observed in the hypothalamus. The pattern of VA, the other rumen-derived trans FA, did not mirror its dietary concentration, and higher values were observed in all tissues except the hypothalamus in rats fed diet B. Moreover, in liver and both adipose tissues, rats on diet C, displayed higher amount than those fed diet A (Table 2).

Table 3 shows that CLA was efficiently desaturated to CD18:3 and elongated to CD20:3 in the liver. A similar pattern was observed in other tissues (data not shown), and despite the higher concentration of CLA in diet B compared to diet C, CD20:3 in the B group was not significantly different from the concentrations found in the C group.

As shown in Table 2, ALA concentrations were significantly increased in diet B compared to diet D in the liver, and in diet B compared to all the other groups in heart; in VAT in B compared to C and D; in SAT in B compared to the A and C groups. No significant differences were detected in plasma, while ALA was not detectable in the hypothalamus.

EPA was not detectable in the hypothalamus, and was lower in the tissues of animals fed vegetable fat compared to those fed meat fats. EPA was significantly increased mainly with diets A and B compared to the other groups (Table 2). Among all the dietary groups, DHA significantly increased in SAT compared to D, while in heart, DHA was significantly higher in A compared to all other groups; no changes were observed in liver, plasma, and VAT. In the hypothalamus, DHA levels were significantly reduced by diet C compared to diet D (Table 2). Interestingly, DHA concentrations seem to vary according to the dietary amount of CLA, rather than in relation to the dietary content of its putative precursor, ALA, in liver, heart, and plasma. The maximum yield of DHA was obtained at the lowest ALA/CLA ratio in the diet (0.3 in A). DHA concentrations, except in the hypothalamus, were higher in group A, though significantly, only in heart (Table 1 and Table 2).

The n-3 HUFA score was significantly increased in tissues of meat-fat-fed rats compared to those fed vegetable fat, particularly with diets A and B, except in the hypothalamus, in which the level increased significantly only with diet A (Table 2). Interestingly, the n-3 HUFA score was higher in the tissues of rats fed diets with high LA/ALA ratios (diets A and B) (see Table 1 and Table 2).

The main SFA concentrations did not change among the groups in liver, heart, SAT, plasma, and hypothalamus, while they were slightly, but significantly, increased in VAT by diets B and C compared to diet D (data not shown).

AIFAI was significantly increased with meat fat diets in liver, heart, and VAT. Specifically, diets A and B induced the highest increase in liver and SAT, while in the heart, A was significantly higher than B, C, and D, while B and C were higher than D. In the hypothalamus, this index was raised only in A compared to the C and D groups, and in plasma in A and B compared to D (Table 4).

### 3.2. Tissue Endocannabinoids and Congeners 

**Figure 1:**
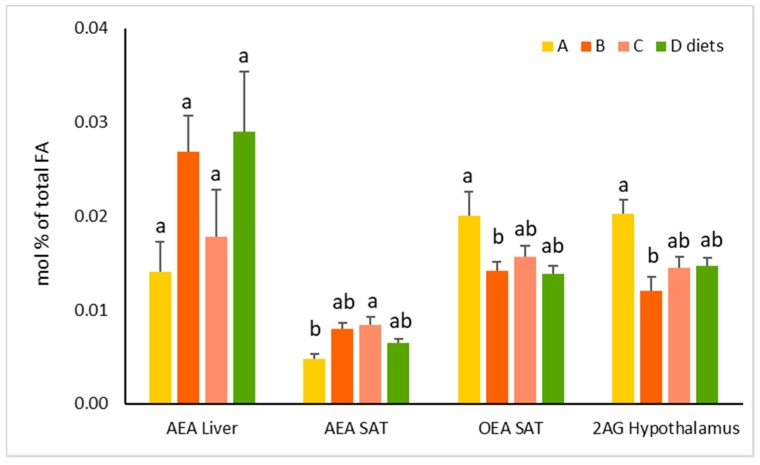
AEA, OEA, and 2-AG in Liver, subcutaneous adipose tissue (SAT), and hypothalamus from rats fed diets A, B, C, or D for 4 wk. Fats in the diets were: (A) enriched in CLA; (B) enriched in ALA and low in CLA; (C) low in ALA and CLA; D) high in LA and trace level of CLA. Values are expressed as mol% of total fatty acids and represent means ± SEMs, *n* = 6/group. Within a tissue, labelled means in a variable without a common superscript letter differ as determined by Tukey’s post hoc test after a significant one-way ANOVA, *p* < 0.05; the maximum value is labeled as ‘a’, the smaller value with difference is marked as ‘b’.

An analysis of EC and congeners, OEA, and PEA, was performed in all tissues except plasma; these compounds were only marginally influenced by diet. Specifically, AEA in SAT was significantly decreased in rats fed diet A compared to those fed diet C, while OEA was significantly increased in A compared to B (Figure 1). In hypothalamus, 2-AG was significantly increased in A compared to B; AEA in liver was slightly reduced with diet A.

## 4. Discussion

The fatty acid composition of ruminant meat is strongly influenced by the diet of animals. In the present study, we compared three kinds of meat fats obtained from lambs under different feeding regimens: meat from light lambs fed only milk from grazing ewes, meat from heavy lambs maintained under a typical intensive feeding regimen based on cereal grains, integrated or not with rolled linseed as a source of ALA. It is well known that meat from suckling lambs is usually rich in CLA, especially when lactating ewes are fed on pasture [41]. The use of linseed in the diet of intensive rearing heavy lambs has been associated with increasing amounts of ALA in intramuscular fat. At the same time, the ratio n-6/n-3 FA is lower in the meat fat from lambs fed diets integrated with linseed compared to feeding regimens based on cereal grains [42].

The results of the present study suggest that the FA composition of dietary fat does not always anticipate its metabolic impact in tissues. This study confirms that the CLA naturally found in ruminant fat is able to significantly increase n-3 HUFA score. In fact, previously, we found that the intake of CLA naturally incorporated into 90 grams of enriched cheese for four weeks, or 50 grams for two months, significantly increased plasma DHA in humans, suggesting that amount and duration are key aspects of CLA intake to induce DHA biosynthesis [38]. In that study, we observed that enriched cheese intake increased PPAR-α gene expression, which is responsible for the induction of key enzymes of peroxisomal β-oxidation [19,48], which is involved in DHA biosynthesis [49]. Our data indicated that irrespective of the matrix, natural CLA is able to increase the n-3 HUFA score. Most of the effects attributed to the n-3 PUFA family are mainly related to dietary EPA and DHA, while ALA seems to have other beneficial effects which are unconnected to its putative property as precursor of EPA and DHA [50]. In fact, the biosynthesis of DHA requires a crucial step in peroxisome for a partial β-oxidation [51]. Moreover, ALA might act as inhibitor of Δ-6-desaturase, which is essential for DHA synthesis [52]. Therefore, any other event that increases desaturase activities and peroxisomal β-oxidation may also favor DHA biosynthesis.

Our results have shown that, in all tissues except in hypothalamus, ALA and CLA incorporation and metabolization is proportional to their concentration in the diet. On the other hand, DHA concentrations changed mostly depending on the relative amount of CLA and only slightly according to their parent availability in the diet. In our earlier human studies, we found that dietary CLA, in a specific, very low range of ALA/CLA ratio (1:3), was able to significantly increase n-3 HUFA biosynthesis [39], while in a previous study, dietary ALA/CLA in a ratio 11/3.2 failed to enhance DHA biosynthesis [53], suggesting that CLA rather than ALA is crucial to enhance DHA biosynthesis. Therefore, on the basis of the data available in the literature, it seems that CLA products could be an unexpected source of DHA. Diet A, enriched in CLA, contains more DHA and EPA than the other diets; however, the concentration of EPA and DHA are extremely low and, for example, while EPA has similar concentration in diets B and C, and about 1/3 lower than diet A, EPA levels in tissues of rats fed diets A and B are in general significantly higher than in tissues of rats fed the diet C. As a matter of fact, the n-3 UFA score was found to be similarly increased in tissues of rats fed diets A and B, irrespective of EPA and DHA differences in the diets.

The plasma n-3 HUFA score is widely used to evaluate the impact of a nutritional treatment on the balance of n-3/n-6 HUFAs [54]. Our data showed that the highest increase in n-3 HUFA score was induced by A and B meat fat diets which were enriched in ALA and CLA (Table 2).

Interestingly, in group A, characterized by high levels of CLA and an ALA/CLA ratio of 0.3, the increase of n-3 HUFA score was mainly attributed to an increase of DHA, while in diet B, it was characterized by a higher ALA/CLA ratio, i.e., 1.5. The n-3 HUFA score increase was due to a greater tissue concentration of EPA. Since data from human studies are usually limited to plasma analyses, we also evaluated changes in FA metabolism in different tissues.

Interestingly, the higher incorporation in tissues of DHA and CLA induced by diet A was associated with a significant increase of OEA and a concomitant decrease of AEA in some tissues like SAT (Figure 1). Dietary CLA supplementation may increase OEA levels in the livers of obese Zucker rats, possibly by activating PPAR-α [55], which may also contribute to the higher DHA biosynthesis via enhanced peroxisomal β-oxidation. These data are in agreement with what we previously found, i.e., that in obese rats, a diet enriched with n-3 HUFA resulted in the reduction of EC biosynthesis as a result of a decrease in their precursor concentration in membrane PLs, which may account for the reduction of ectopic fat and inflammatory mediators [43], and imply that DHA and CLA may exert a direct effect on EC and the biosynthesis of congeners. Conversely, diet B, with a relatively high dietary ratio ALA/CLA, may result in a lower PPAR-α activation, which may explain the significantly higher accumulation of VA in tissues due to a reduced peroxisomal β-oxidation. In fact, it has been demonstrated that trans FAs are preferentially β-oxidized in peroxisomes [56], which are regulated by PPAR-α [57].

The n-3 HUFA score showed a pattern similar to CLA or VA in liver and plasma, but not in the hypothalamus, as expected, which appeared to be more resistant to FA profile modification by dietary means, with early administration in life and duration of exposure, as well as dietary concentrations, being key factors in the detection of significant alterations [58]. Nevertheless, in hypothalamus, we found a significant increase in the AA concentration with the vegetable oil-based diet D, rich in LA, a precursor of AA; meanwhile, with diet A, enriched in CLA, we found an increase in the n-3 HUFA score. Our data suggest that the n-3 HUFA score change in the hypothalamus was not due to an increased biosynthesis of DHA and EPA in this tissue, but rather, to an increased transportation of these n-3 PUFAs from plasma. One can speculate that changes of FAs in peripheral tissue can directly influence FA concentrations in specific brain areas.

These modest changes in the hypothalamus were in the order of 15–20%, and may not be sufficient to exert significant effects on feeding behavior. Accordingly, the increase of 2-AG found in the hypothalamus of rats fed diet A was not associated with changes in food intake.

We previously demonstrated that CLA passes the blood brain barrier [59]; in the present experimental setting dietary, the CLA level was probably too low to be incorporated into the hypothalamus. Future studies should aim at evaluating whether dietary meat fat higher in CLA and/or longer feeding periods are able to modify hypothalamus CLA levels and influence feeding behavior through PPAR-α activation.

Another remarkable feature which may influence the n-3 HUFA score is the LA/ALA ratio, based on the concept that diets high in LA would inhibit the synthesis of n-3 HUFA by simple competitive inhibition [29,30]. In chickens, it has been found that when the LA/ALA ratio in the diet was above 5, liver PLs were rich in AA and poor in EPA; meanwhile, when the ratio dropped below 5, there was an exchange of AA for EPA [60]. The DHA status increased with a dietary level of ALA of around 1%en in rats, after which DHA accumulation was inhibited and then declined [60]. The LA/ALA ratio in our experimental diets was 2.1 (ALA 0.09%en) in A, 2.2 (ALA 0.21%en) in B, 7.0 (ALA 0.081%en) in C, and 22.0 (ALA 0.1%en) in D (Table 1). We found a significantly reduced n-3 HUFA score with C, and particularly with D in all tissues, while in hypothalamus, as reported, we observed an increase for diet A.

Since obesity is regarded as a low-grade chronic inflammatory condition characterized by increased proinflammatory cytokines in the white adipose tissue [61], it had been suggested that diets that can enhance n-3 PUFA could reduce the synthesis of PGE2 and enhance the production of PG involved in the resolution of inflammatory disorders [34]. We found that AIFAI index was significantly increased in the tissues of obese rats fed meat fat diets compared to obese rats fed vegetable fat diets (Table 4).

Interestingly, as observed in hypothalamus and in the other tissues, our data revealed that AA in SAT was significantly decreased with diets based on meat fat compared to those with vegetable fat. It is possible that increased CLA intake may interfere with the further metabolism of LA. We have previously seen that even though there was no perturbation in tissue LA, LA metabolites (including 18:3n-6, DGLA, and in particular AA) were consistently depressed in tissues by up to 1% CLA in the diet [11]. Consequently, CLA might further enhance the AIFAI index probably by inducing a decrease in AA and an increase in DHA biosynthesis. It is possible that CLA, or its relative metabolites, might differentially modulate the distribution of AA in various PLs [62], competing with AA for incorporation; therefore, this scenario may affect the eicosanoid signaling mechanism.

The consumption of meat fat rich in CLA-ALA resulted in significantly increased accumulation of DHA and depression of AA synthesis, which may have therapeutic potential to ameliorate clinical symptoms and complications that are secondary to the excessive production of proinflammatory mediators. Our data clearly indicate that metabolic changes by dietary FAs seem to be tissue specific and affected by other factors such as background diet, energy and lipid metabolism. However, in our model of obesity, we didn’t find any changes in parameters of metabolic syndrome such as dyslipidemia or fatty liver, probably due to the relative short-term feeding period or to the relatively low CLA concentration.

## 5. Conclusions

Our data put in evidence that the feeding system of livestock may play an important role in modulating the effect of meat fat on lipid metabolism, as some FAs, like CLA and ALA, improve the tissue FA profile, as shown with the increased n-3 HUFA score. While not being comparable to the direct intake of EPA and DHA through fish products, it seems that meat that is naturally enriched with CLA could be an unexpected source of DHA, provided that a specific ratio of FAs in the pool of total FAs is respected.

These results are promising, especially regarding individuals for whom the intake of fish products is quite low, i.e., far below the recommended daily dose. Future studies are envisaged to evaluate whether dietary fat of different origin and composition is able to modify these parameters in humans.

## Figures and Tables

**Table 1:** Principal fatty acids in experimental diets. ^1^

FA	A ^2^	B ^2^	C ^2^	D ^2^
	g/kg diet			
14:0	4.3	2.6	2.4	0.3
16:0	13.1	11.6	12.6	5.9
18:0	8.6	9.5	10.8	1.4
VA	1.4	1.0	0.6	-
OA	22.2	19.7	21.6	33.7
LA	0.8	2.0	2.3	13.1
ALA	0.4	0.9	0.3	0.6
CLA	1.30	0.61	0.41	0.03
EPA	0.09	0.06	0.05	-
DHA	0.13	0.05	0.05	-
SFA	27.9	25.4	27.7	7.6
UFA	30.8	32.9	31.2	47.3
n-6/n-3	1.1	1.1	4.2	22.0
ALA/CLA	0.3	1.5	0.8	22.2
LA/ALA	2.2	2.2	7.0	22.0
total FA	58.7	58.3	58.8	54.9

^1^ Diets were AIN-93G by Harlan; standard fat formulation was substituted with experimental fats. ^2^ Fats in the diets were: (A) enriched in conjugated linoleic acid (CLA); (B) enriched in α-linolenic acid (ALA) and low in CLA; (C) low in ALA and CLA; (D) high in linoleic acid (LA) and trace levels of CLA. Vaccenic acid (VA), oleic acid (OA), eicosapentaenoic acid (EPA), docosahexaenoic acid (DHA), saturated fatty acid (SFA), unsaturated fatty acid (UFA).

**Table 2:** FA concentrations in obese Zucker rats fed diets A, B, C, or D ^1^ for 4 wk.

Diet Group	Liver (nmol/g)	Heart (nmol/g)	VAT (nmol/g)	SAT (mol/g)	Plasma (nmol/mL)	Hypothalamus (nmol/g)
ALA						
A1	898.7 ± 128.3 ^ab^	102.5 ± 11.8 ^a^	23926.7 ± 1248.4 ^ab^	24589.5 ± 1560.4 ^b^	97.7 ± 13.5 ^a^	ND
B1	1190.2 ± 100.1 ^b^	208.6 ± 18.9 ^b^	27737.5 ± 978.7 ^a^	31367.0 ± 1096.6 ^a^	112.1 ± 17.2 ^a^	ND
C1	969.6 ± 61.6 ^ab^	126.2 ± 10.1 ^a^	22779.2 ± 1710.2 ^b^	26599.3 ± 1326.9 ^b^	103.1 ± 7.1 ^a^	ND
D1	596.8 ± 163.3 ^a^	103.7 ± 15.9 ^a^	20194.1 ± 1017.2 ^b^	25711.5 ± 1746.5 ^ab^	80.8 ± 5.2 ^a^	ND
EPA						
A1	541.0 ± 92.3 ^a^	59.4 ± 3.3 ^a^	407.8 ± 35.6 ^ab^	506.8 ± 22.6 ^ab^	64.5 ± 2.9 ^a^	ND
B1	555.9 ± 70.6 ^a^	61.9 ± 2.9 ^a^	495.9 ± 38.3 ^a^	605.9 ± 42.1 ^a^	65.5 ± 7.2 ^a^	ND
C1	377.1 ± 34.0 ^ab^	42.7 ± 1.0 ^b^	284.3 ± 17.9 ^bc^	386.6 ± 20.6 ^bc^	55.1 ± 3.1 ^ab^	ND
D1	185.1 ± 59.7 ^b^	22.2 ± 1.1 ^c^	169.0 ± 21.8 ^c^	262.1 ± 28.5 ^c^	32.3 ± 2.0 ^b^	ND
DHA						
A1	8242.1 ± 407.5 ^a^	4318.2 ± 161.8 ^a^	2604.9 ± 372.5 ^a^	2403.9 ± 111.3 ^a^	480.6 ± 16.9 ^a^	11791.5 ± 881.7 ^ab^
B1	8101.5 ± 514.4 ^a^	3709.7 ± 115.7 ^b^	2124.2 ± 187.6 ^a^	2014.8 ± 134.8 ^ab^	428.3 ± 48.9 ^a^	11687.5 ± 349.5 ^ab^
C1	7752.8 ± 394.0 ^a^	3506.4 ± 95.7 ^bc^	2307.0 ± 189.3 ^a^	1986.7 ± 106.9 ^ab^	439.4 ± 26.1 ^a^	11331.8 ± 369.2 ^b^
D1	6720.0 ± 671.9 ^a^	3068.7 ± 201.6 ^c^	1500.0 ± 264.6 ^a^	1664.0 ± 23.0 ^b^	410.4 ± 45.2 ^a^	13612.8 ± 519.5 ^a^
n-3 HUFA score						
A1	32.0 ± 0.8 ^a^	34.6 ± 0.3 ^a^	22.8 ± 2.4 ^ab^	23.7 ± 1.0 ^a^	22.8 ± 0.91 ^a^	49.8 ± 0.7 ^a^
B1	30.3 ± 0.6 ^a^	31.9 ± 1.1 ^a^	23.5 ± 0.8 ^a^	22.2 ± 0.9 ^a^	21.2 ± 0.72 ^ab^	48.9 ± 0.1 ^ab^
C1	28.1 ± 0.2 ^b^	32.0 ± 1.1 ^a^	19.3 ± 1.0 ^b^	18.9 ± 0.7 ^b^	20.2 ± 0.33 ^b^	48.3 ± 0.2 ^b^
D1	24.4 ± 0.23 ^c^	25.1 ± 0.7 ^b^	14.2 ± 1.1 ^c^	12.8 ± 0.7 ^c^	16.2 ± 0.02 ^c^	47.6 ± 0.2 ^b^
AA						
A1	19396.2 ± 994.3 ^a^	9694.9 ± 74.9 ^a^	8253.3 ± 277.1 ^a^	8558.3 ± 319.4 ^b^	2058.7 ± 57.1 ^a^	8670.8 ± 648.7 ^b^
B1	20722.6 ± 1212.4 ^a^	9762.2 ± 117.0 ^a^	8138.1 ± 581.0 ^a^	8820.2 ± 457.9 ^b^	2089.9 ± 152.1 ^a^	9066.6 ± 252.8 ^b^
C1	21505.6 ± 1048.2 ^a^	9638.7 ± 102.4 ^a^	9364.8 ± 645.9 ^a^	10015.1 ± 418.8 ^b^	2237.5 ± 90.7 ^a^	9036.5 ± 244.4 ^b^
D1	22810.6 ± 2082.8 ^a^	9885.1 ± 236.5 ^a^	10188.2 ± 889.6 ^a^	12291.9 ± 566.0 ^a^	2575.2 ± 213.2 ^a^	11128.7 ± 436.4 ^a^
CLA						
A1	622.6 ± 66.7 ^a^	84.1 ± 10.4 ^a^	19892.6 ± 473.5 ^a^	17208.7 ± 931.2 ^a^	58.9 ± 7.3 ^a^	13.4 ± 0.5 ^a^
B1	416.2 ± 27.9 ^b^	75.8 ± 8.7 ^a^	12108.4 ± 396.1 ^b^	11697.7 ± 424.9 ^b^	35.6 ± 5.3 ^b^	17.7 ± 1.3 ^a^
C1	280.7 ± 17.4 ^c^	43.2 ± 2.9 ^b^	8218.0 ± 577.4 ^c^	7911.8 ± 251.0 ^c^	29.1 ± 1.8 ^bc^	11.7 ± 1.5 ^a^
D1	58.2 ± 11.1 ^d^	12.7 ± 2.8 ^c^	1585.7 ± 171.0 ^d^	1669.5 ± 181.7 ^d^	10.9 ± 1.8 ^c^	11.2 ± 4.8 ^a^
VA						
A1	529.7 ± 109.7 ^b^	274.1 ± 5.7 ^b^	14613.7 ± 200.3 ^b^	12199.2 ± 968.5 ^b^	76.6 ± 5.79 ^a^	656.8 ± 152.5 ^a^
B1	1248.4 ± 249.9 ^a^	389.7 ± 14.1 ^a^	35216.2 ± 384.0 ^a^	30441.6 ± 1602.2 ^a^	122.5 ± 17.93 ^a^	693.5 ± 54.7 ^a^
C1	732.1 ± 151.1 ^bc^	219.6 ± 11.1 ^c^	19793.3 ± 1407.8 ^bc^	17789.3 ± 1032.3 ^bc^	96.3 ± 6.52 ^ab^	707.0 ± 47.3 ^a^
D1	214.7 ± 82.3 ^bd^	124.8 ± 5.6 ^d^	2962.6 ± 342.8 ^d^	2844.9 ± 212.1 ^d^	47.5 ± 7.87 ^b^	744.3 ± 14.3 ^a^

Values are means ± SEMs, *n* = 6/group. Within tissue, labelled means in a variable without a common superscript letter differ, as determined by Tukey’s post hoc test after a significant one-way ANOVA, *p* < 0.05; the maximum value is labeled as ‘a’, the smaller value with difference is marked as ‘b’, the smaller value than ‘b’ with difference is marked as ‘c’, and the smallest value with difference is marked as ‘d’. ND not detected. Subcutaneous (SAT), visceral adipose tissue (VAT); arachidonic acid (AA), n-3 highly-unsaturated fatty acids (n-3 HUFA score). ^1^ Fats in the diets were: (A) enriched in CLA; (B) enriched in ALA and low in CLA; (C) low in ALA and CLA; (D) high in LA and trace level of CLA.

**Table 3:** CLA and its metabolites in liver of obese Zucker rats fed diets A, B, C, or D for 4 wk^1^.

	nmol/g Liver		
Diet Groups	CLA	CD18:3	CD20:3
A^1^	622.6 ± 66.7 ^a^	34.1 ± 4.5 ^a^	59.1 ± 3.1 ^a^
B^1^	416.2 ± 27.9 ^b^	28.0 ± 4.4 ^a^	28.6 ± 1.8 ^b^
C^1^	280.7 ± 17.4 ^c^	10.3 ± 1.5 ^b^	24.2 ± 1.9 ^b^
D^1^	58.2 ± 11.1 ^d^	6.7 ± 1.3 ^b^	7.7 ± 0.6 ^c^

Values are means ± SEMs, *n* = 6/group. Within a variable, labelled means without a common superscript letter differ as determined by Tukey’s post hoc test after a significant one-way ANOVA, *p* < 0.05; the maximum value is labeled as ‘a’, the smaller value with difference is marked as ‘b’, the smaller value than ‘b’ with difference is marked as ‘c’, and the smallest value with difference is marked as ‘d’. Conjugated dienes (CD). ^1^ Fats in the diets were: (A) enriched in CLA; (B) enriched in ALA and low in CLA; (C) low in ALA and CLA; (D) high in LA and trace level of CLA.

**Table 4:** Anti-inflammatory FA index (AIFAI) obtained as (EPA + DHA + DGLA)/AA in tissues of obese Zucker rats fed diets A, B, C, or D for 4 wk ^1^.

	Anti-Inflammatory Index				
Diet Groups	Heart	VAT	SAT	Plasma	Hypothalamus
A1	52.8 ± 1.3 ^a^	78.8 ± 6.7 ^a^	84.3 ± 5.3 ^a^	31.3 ± 1.9 ^a^	139.9 ± 6.2 ^a^
B1	49.1 ± 0.7 ^b^	70.3 ± 2.2 ^a b^	77.5 ± 4.8 ^a^	28 ± 1.7 ^a^	131.5 ± 0.6 ^ab^
C1	44 ± 0.3 ^c^	64.8 ± 1.2 ^b^	62.2 ± 2.0 ^bc^	26.2 ± 0.7 ^ab^	127.6 ± 1.2 ^b^
D1	34 ± 0.6 ^d^	45.4 ± 1.2 ^c^	46.4 ± 5.7 ^c^	20.6 ± 0.8 ^b^	124.1 ± 1.2 ^b^

Values are means ± SEMs, *n* = 6/group. Within a tissue, labelled means without a common superscript letter differ as determined by Tukey’s post hoc test after a significant One-way ANOVA, Tukey’s multiple comparisons test, *p* < 0.05; the maximum value is labeled as ‘a’, the smaller value with difference is marked as ‘b’, and the smallest value with difference is marked as ‘c’. Subcutaneous (SAT) and visceral adipose tissue (VAT). ^1^ Fats in the diets were: (A) enriched in CLA; (B) enriched in ALA and low in CLA; (C) low in ALA and CLA; (D) high in LA and trace level of CLA.
